# Cannabinoid, melanocortin and opioid receptor expression on *DRD1* and *DRD2* subpopulations in rat striatum

**DOI:** 10.3389/fnana.2014.00014

**Published:** 2014-03-26

**Authors:** Ralph J. A. Oude Ophuis, Arjen J. Boender, Andrea J. van Rozen, Roger A. H. Adan

**Affiliations:** ^1^Department of Translational Neuroscience, Brain Center Rudolf Magnus, University Medical Center UtrechtUtrecht, Netherlands; ^2^Department of Reproductive Medicine and Gynaecology, University Medical Center UtrechtUtrecht, Netherlands

**Keywords:** striatum, GPCR, opioid, cannabinoid, melanocortin, dopaminergic, FISH

## Abstract

The striatum harbors two neuronal populations that enable action selection. One population represents the striatonigral pathway, expresses the dopamine receptor D1 (DRD1) and promotes the execution of motor programs, while the other population represents the striatopallidal pathway, expresses the dopamine receptor D2 (DRD2) and suppresses voluntary activity. The two populations integrate distinct sensorimotor, cognitive, and emotional information streams and their combined activity enables the selection of adaptive behaviors. Characterization of these populations is critical to the understanding of their role in action selection, because it aids the identification of the molecular mechanisms that separate them. To that end, we used fluorescent *in situ* hybridization to quantify the percentage of striatal cells that (co)express dopaminergic receptors and receptors of the cannabinoid, melanocortin or opioid neurotransmitters systems. Our main findings are that the cannabinoid 1 receptor is equally expressed on both populations with a gradient from dorsal to ventral striatum, that the opioid receptors have a preference for expression with either the *DRD1* or *DRD2* and that the melanocortin 4 receptor (*MC4R)* is predominantly expressed in ventral parts of the striatum. In addition, we find that the level of *MC4R* expression determines its localization to either the *DRD1* or the *DRD2* population. Thereby, we provide insight into the sensitivity of the two dopaminoceptive populations to these neurotransmitters and progress the understanding of the mechanisms that enable action selection.

## Introduction

To ensure survival, an organism needs to select adaptive behaviors from its behavioral repertoire. One neural network that is involved in action selection is the basal ganglia, which consists of several subcortical structures (Mogenson et al., 1980). The input nucleus of the basal ganglia is the striatum (Smith et al., 1994; Kincaid et al., 1998; Bolam et al., 2000), a brain area in which GABAergic projection neurons make up for more than 95% of the cellular identity (Kreitzer, 2009). These medium spiny neurons (MSNs) can be divided into two classes, based on the expression of either the dopamine receptor D1 (D1R) or the dopamine receptor D2 (D2R) (Gerfen et al., 1990; Le Moine and Bloch, 1995).

Both cellular populations influence the activity of the output nucleus of the basal ganglia; the internal globus pallidus/substantia nigra reticular part (GPi/SNpr) complex. The D1R population sends inhibitory projections to the Gpi/SNpr complex directly, while the D2Rpopulation activates the GPi/SNpr complex indirectly, via the external globus pallidus and the subthalamic nucleus (STN) (Gerfen, 1992; Kawaguchi, 1997; Gerfen and Surmeier, 2011). Activity of the GPi/SNpr is thought to exert control over voluntary movement by sending inhibitory signals to thalamic and brain stem nuclei. Much attention has been directed toward the existence of the antagonistic D1R and D2R populations that drive GPi/SNpr activity (Alexander et al., 1986; Graybiel, 2000), because they are the physical representation of an important concept in the neurosciences: the “go-no go” pathway. In this model, the D1R population represents “go” as it inhibits the GPi/SNpr, while the D2R represents “no go” as it activates the GPi/SNpr. Activity of the D1R population is thought to promote the execution of motor programs, while activation of the D2R population suppresses voluntary movement (Gerfen and Surmeier, 2011). While it is far from clear how these two populations work together to select adaptive behaviors, combined activity of these populations has been suggested to enable the execution of one particular motor program, by simultaneous suppression of competing motor programs (Mink, 2003; Cui et al., 2013).

In search of the molecular mechanisms that separate the two MSN populations and enable the selection of adaptive behaviors, the focus should not be limited to the dopaminergic system only, as dopaminergic receptors are not the only G-protein coupled receptors (GPCR) whose expression is limited to either of the two MSN populations. In addition, some neuropeptides are differentially expressed in the two MSN populations as well: D1R neurons exclusively express the acetylcholine muscarinic receptor 4 and substance P (Gerfen and Young, 1988; Bernard et al., 1992; Ince et al., 1997), while expression of the adenosine receptor 2a (A2aR) and enkephalin is limited to the D2R population (Gerfen and Young, 1988; Schiffmann et al., 1991). Moreover, it should be noted that the striatum can be divided into separate functional regions that process distinct information streams and are aligned to a gradient that is organized in a dorsolateral to ventromedial fashion (Voorn et al., 2004). Dorsolateral regions are primarily involved in the processing of sensorimotor information (Albin et al., 1989; Graybiel et al., 1994; Chang et al., 2002; Haber, 2003; Balleine et al., 2007; Durieux et al., 2012), central regions mediate cognitive functions such as procedural learning and working memory (Phillips and Carr, 1987; Jog et al., 1999), while ventromedial parts are associated with the integration of motivational state (Kelley and Domesick, 1982; Kelley et al., 1982; McGeorge and Faull, 1989; Cardinal et al., 2002). As separate striatal regions process different information streams, it is to be expected that they show differences in cellular identity, which could arise through the innervation of distinct neurotransmitter systems. Therefore, the location and instances of (co)expression of their receptors can serve to mark differences in cellular identity and aids the understanding of the molecular mechanisms that enable striatal functioning.

To that end, we here used fluorescent *in situ* hybridization (FISH) to investigate the (co)expression of dopamine receptor D1 (*DRD1*) and the dopamine receptor D2 (*DRD2*) mRNAs with transcripts of the GPCRs of the cannabinoid, melanocortinergic and opioid neurotransmitter systems. We quantified expression levels in five different areas, the lateral and medial caudate putamen (lCPu, mCPu), the nucleus accumbens core and shell (AccC and AccSh) and the olfactory tubercle (OT), which is thought to share functionality with the nucleus accumbens (Ikemoto, 2007). These neurotransmitter systems are known to modulate the activity of MSNs in conjunction with dopaminergic neurotransmission and visualization of their receptors might mark cellular subpopulations in the striatum. In doing so, we provide insight into the cellular architecture of the striatum and progress the understanding of its role in action selection.

## Materials and methods

### Animals

Adult male Wistar rats (Charles-River, Erkrath, Germany) were used (*n* = 2). The animals were housed individually (378 × 217 × 180 cm) in a controlled environment under a 12:12 light/dark cycle with lights on at 0700 h. The experiments were approved by the Animal Ethics Committee of the University Medical Center Utrecht, according to Dutch legislation.

### Fish probes

The probe sets used to detect the desired rat mRNAs are designed by Panomics (Santa Clara, USA), using published sequences (see Table 1). Blast searches were performed to avoid large homologous sequences of undesired genes and guarantee probe specificity. The probe sets consist of about 20 probe pairs, with each probe having a length of 20–25 nucleotides. The probes within each pair are designed to hybridize at adjacent regions of the target mRNA, allowing the hybridization of a preamplification oligo (Panomics) that spans the hybridized probe pair, thus ensuring signal specificity. This specific signal was further amplified by hybridization of amplification oligo's (Panomics) and visualized by label oligo's (Panomics), resulting in an amplification of up to 500 times for low abundant mRNAs. In addition, probe pairs were designed to hybridize at adjacent sections, covering the entire region that is mentioned in Table 1. Other probes were designed to hybridize to small regions within the desired gene sequence that share homology with undesired gene sequences. These probes did not allow hybridization of preamplification oligo's, which ensured specificity of the amplified signal.

**Table 1:** **Regions and accession numbers used for synthesis of the FISH probes**.

**Gene**	**Accession number**	**Region**
*DRD1*	NM_012546	29-2164
*DRD2*	NM_012547	410-1488
*CB1R*	NM_012784	175-1120
*MC4R*	NM_013099	231-1136
*MC3R*	NM_001025270	198-1076
*MOR*	NM_013071	226-1228
*DOR*	NM_012617	120-1095
*KOR*	NM_017167	133-1162
*A2aR*	NM_053294	504-1611
*ENK*	NM_017139	21-1036
*SubP*	NM_012666	89-940

### Fish procedure

After decapitation, brains were isolated and immediately frozen on dry ice. Coronal sections (16 μm) were made with a Leica CM 3050 cryostat between 1.38 and 1.70 mm anterior to bregma (Leica, Wetzlar, Germany). First, sections were post-fixed in 4% paraformaldehyde for 10 min, washed in phosphate buffered saline (PBS) and acetylated for 10 min. After washing, sections were subsequently incubated for 2 h in prehybridization mix (50% deionized formamide, 5× SSC, 5× Denhardt's solution, 250 μg/ml tRNA baker's yeast, 500 μg/ml sonicated salmon sperm DNA final concentrations in MilliQ). Next, sections were incubated overnight at 40°C in 120 μl hybridization mix (Panomics) containing the probe sets (*DRD1* 1:33, *DRD2* 1:33, other probes 1:50) and washed. Subsequently, sections were incubated for 1.5 h at 40°C in 120 μl PreAmplifier Mix (Panomics) containing preamplification oligo's (PreAmp TYPE4 1:20, PreAmp TYPE 6 1:50, PreAmp TYPE 8 1:33) and washed. Then, sections were incubated for 1.5 h at 40°C in 120 μl Amplifier Mix (Panomics) containing amplification oligo's (Amp TYPE4 1:20, Amp TYPE 6 1:50, Amp TYPE 8 1:33) and washed. Next, sections were incubated for 1.5 h at 40°C in 120 μl in Label Probe Mix (Panomics) containing label oligo's (LP TYPE4 1:20, LP TYPE 8 1:33, LP TYPE 6 1:50). After washing, sections were incubated in 750 μl PBS supplemented with 4',6-diamidino-2-phenylindole (DAPI) (6.7 μg/ml, Sigma Aldrich, St. Louis, USA) for 5 min. Finally, sections were washed and embedded in Mowiol.

### Image acquisition

Images were obtained with a Zeiss AxioScope A1 microscope (Carl Zeiss, Jena, Germany) equipped with Chroma filter sets (Chroma, Bellows Falls, USA) and Zeiss AxioVision Rel. 4.8 acquisition software. DAPI was acquired using the 31000v2 filter block of Chroma. The Chroma FITC filter block 410001 was used to acquire the 488 nm conjugated TYPE4 label. The 550 nm conjugated TYPE8 label was acquired using a Chroma TRITC 41002b filter block containing a narrowband excitation filter. A custom Chroma Cy5 infrared filter was used for the acquisition of the 650 nm conjugated TYPE6 label. This label was excited at 650 nm using an HQ650/45x filter (Chroma) and light was directed by a Q680LP dichroic mirror (Chroma) through a HQ690LP emission filter (Chroma). Images were processed and analyzed using ImageJ 1.43r software.

### Data analysis

The (co)expression of the mRNA transcripts of the different GPCRs and neuropeptides was quantified in five different areas of the striatum: the lateral caudate putamen (lCPu), the medial caudate putamen (mCPu), the nucleus accumbens core (AccC), the nucleus accumbens shell (AccSh) and the olfactory tubercle (OT). Four images (300 × 220 μm) per striatal area were used for quantification of a certain mRNA with the *DRD1* and *DRD2* transcripts. Separate cells were identified on the basis of nuclear DAPI staining and were counted as expressing a certain mRNA if one or more fluorescent dots were present in, or in close vicinity of (defined as a circle with twice the diameter of the DAPI staining) the area of DAPI staining.

The percentage of cells expressing a certain mRNA was determined by dividing the amount of cells expressing a certain mRNA by the total amount of DAPI stained nuclei (= number of cells expressing a certain mRNA/the total number of DAPI stained nuclei^*^100). Statistical analyses were done by conduction of the non-parametric Kruskal–Wallis tests, with significance levels set at α = 0.05, to determine significant differences in levels of expression per striatal area in SPSS 20 for Windows (IBM, USA). The Kruskal–Wallis test was used because the small sample sizes did not allow for any inferences on the underlying distributions.

The percentage of cells expressing *DRD1* mRNA and another mRNA was determined by dividing the amount of cells positive for *DRD1* and another mRNA by the total amount of *DRD1* positive cells (number of cells expressing *DRD1* and another mRNA/total number of cells expressing DRD1^*^100). An identical approach was used for calculation of the percentage of *DRD2* expressing cells that also expressed another mRNA. Because of the small sample sizes, the preference for expression of a certain mRNA with transcripts of either *DRD1* or *DRD2* were determined by conduction of non-parametric Mann–Whitney U tests in SPSS 20 for Windows (IBM, USA). For these tests, results from separate striatal areas were combined, as individual tests for each separate striatal area would necessitate corrections for multiple comparisons and essentially yield comparable results.

To determine if cells were either high- or low-expressing, the number of fluorescent dots were counted and cells were arbitrarily classified as low expressing cells if they expressed less than 30 dots and classified as high expressing in all other instances.

## Results

### Coexpression of *DRD1* and *DRD2* with *A2aR*, *ENK* or *SubP*

**Figure 1:**
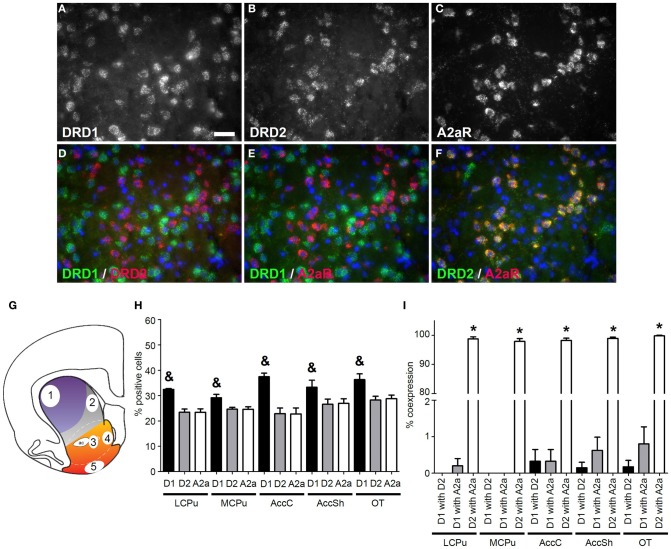
**Coexpression of the adenosine 2a receptor with both *DRD1* and *DRD2*. (A–F)** Example of staining in the lateral caudate putamen (lCPu). Nuclei were stained using 4',6-diamidino-2-phenylindole (DAPI) (blue). Clear expression for the dopamine receptor D1 (*DRD1*) **(A)**, the dopamine receptor D2 (*DRD2*) **(B)** and the adenosine 2a receptor (*A2aR*) **(C)**. White bar, 25 μm. **(D,E)** Virtually no coexpression was found for *DRD1* and *DRD2* and for *DRD1* and *A2aR*. **(F)** Clear coexpression was found for *DRD2* and *A2aR*. **(G)** Quantification of expression and coexpression was performed in several regions of the striatum: 1 = lCPu, 2 = medial caudate putamen (mCPu), 3 = nucleus accumbens core (AccC), 4 = nucleus accumbens shell (AccSh), 5 = olfactory tubercle (OT). Picture adapted from Voorn et al. (2004). **(H)** Quantification of the expression of *DRD1*, *DRD2* and *A2aR*. Bars represent the mean percentage (+s.e.m.) of DAPI stained nuclei that are positive for *DRD1, DRD2* or *A2aR*, where “&” indicate a significant difference of *p* < 0.05 in the mean percentage of *DRD1* positive cells compared to *DRD2* positive cells (as determined by the Mann–Whitney U test). **(I)** Quantification of coexpression of *DRD1*, *DRD2*, and *A2aR*. Bars represent the mean percentage (+s.e.m.) of cells expressing *DRD1* also expressing *DRD2*, the percentage of *DRD1* expressing cells also expressing *A2aR* and the percentage of *DRD2* expressing cells also expressing *A2aR*, where “^*^” indicates a significant difference of *p* ≤ 0.05 in the percentage of cells that coexpress *DRD1* and *A2aR* compared to cells that coexpress *DRD2* and *A2aR* (as determined by the Mann–Whitney U test).

**Figure 2:**
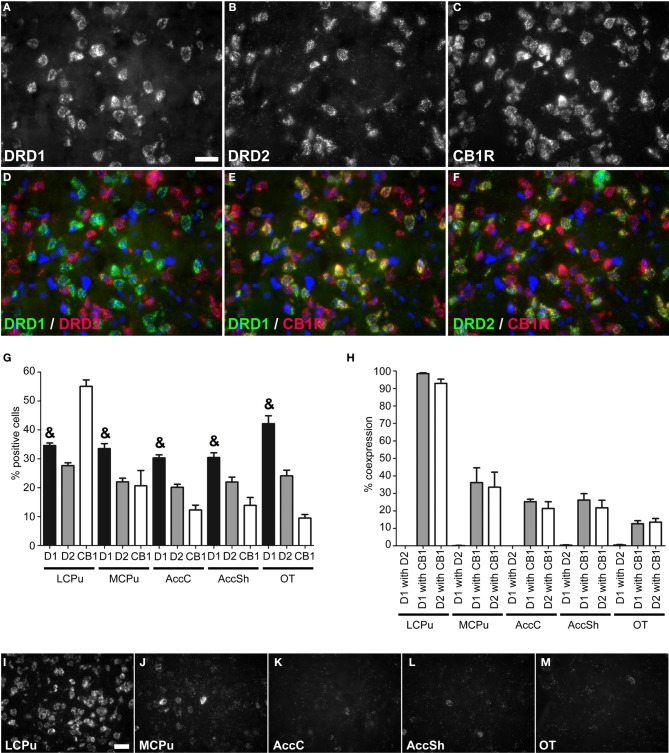
**Coexpression of the cannabinoid receptor 1 with both *DRD1* and *DRD2*. (A–F)** Example of staining in the lCPu. Nuclei were stained using DAPI (blue). Clear expression for the *DRD1* **(A)**, *DRD2* **(B)** and cannabiniod 1 receptor (*CB1R*) **(C)**. White bar, 25 μm. **(D)** No coexpression found for *DRD1* and *DRD2*. **(E,F)** Clear coexpression for *DRD1* and *CB1R* and *DRD2* and *CB1R*. **(G)** Quantification of the expression of *DRD1*, *DRD2* and *CB1R.* Bars represent the mean percentage (+s.e.m.) of DAPI stained nuclei that are positive for *DRD1, DRD2*, or *CB1R*, where “&” indicates a significant difference of *p* < 0.05 in the mean percentage of *DRD1* positive cells compared to *DRD2* positive cells (as determined by the Mann–Whitney U test). **(H)** Quantification of coexpression of *DRD1*, *DRD2*, and *CB1R*. Bars represent the mean percentage (+s.e.m.) of cells expressing *DRD1* also expressing *DRD2*, the percentage of *DRD1* expressing cells also expressing *CB1R* and the percentage of *DRD2* expressing cells also expressing *CB1R*. **(I–M)** Example pictures of *CB1R* expression in the five regions defined within the striatum. White bar, 30 μm.

**Figure 3:**
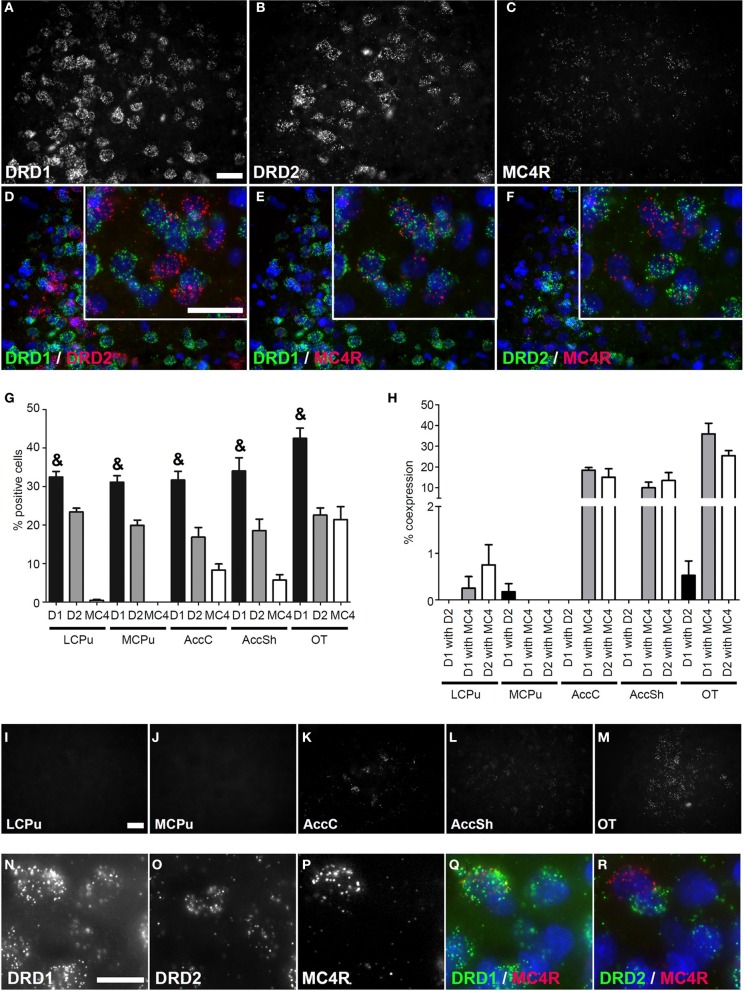
**Coexpression of the melanocortin 4 receptor with both *DRD1* and *DRD2*. (A–F)** Example of staining in the OT. Nuclei were stained using DAPI (blue). Clear expression for the *DRD1* **(A)**, *DRD2* **(B)**, and modest expression for melanocortin 4 receptor (*MC4R*) **(C)**. Enlargements of cells are depicted expressing both *DRD1* and *MC4R*, or both *DRD2* and *MC4R*. White bar, 25 μm. **(D)** No coexpression found for *DRD1* and *DRD2*. **(E,F)** Clear coexpression for *DRD1* and *MC4R* and *DRD2* and *MC4R*. White bar, 25 μm. **(G)** Quantification of the expression of *DRD1*, *DRD2*, and *MC4R.* Bars represent the mean percentage +s.e.m. of DAPI stained nuclei that are positive for *DRD1, DRD2*, or *MC4R*, where “&” indicates a significant difference of *p* < 0.05 in the mean percentage of *DRD1* positive cells compared to *DRD2* positive cells (as determined by the Mann–Whitney U test). **(H)** Quantification of coexpression of *DRD1*, *DRD2*, and *MC4R*. Bars represent the mean percentage (+s.e.m.) of cells expressing *DRD1* also expressing *DRD2*, the percentage of *DRD1* expressing cells also expressing *MC4R* and the percentage of *DRD2* expressing cells also expressing *MC4R*. **(I–M)** Example pictures of *MC4R* expression in the five regions defined within the striatum. White bar, 30 μm. **(N–R)** Cells expressing high amounts of *MC4R* are found in the *DRD1* population. White bar, 10 μm.

We quantified the coexpression of genes that are known to segregate to either the D1R or D2R population (see Table 2). For the D1R population, this entailed quantification of coexpression of *DRD1* mRNAs with mRNAs of the precursor to substance P, protachykinin-1 (*SubP)*. For the D2R population this entailed quantification of coexpression of *DRD2* mRNAs with mRNAs of the genes *A2aR* and the precursor to the opioid neuropeptide enkephalin, proenkephalin (*ENK*). *DRD1* was virtually not expressed in any *DRD2* expressing cell, as only 0.13 ± 0.08% of *DRD1* expressing cells also expressed *DRD2* (*z* = −6.054, *p* < 0.001) (Figures 1A,D,I, 2D and 3D). As expected, *A2aR* showed a preference for coexpression with *DRD2* (*z* = −5.553, *p* < 0.001) (Figures 1B,C,E,F,I), as 0.39 ± 0.15% of *DRD1* expressing cells expressed *A2aR*, while 98.72 ± 0.33% of cells positive for *DRD1* expressed *A2aR*. Specificity was also observed for expression of *DRD2* with *ENK* (*z* = −5.425, *p* < 0.001) (Supplementary Figures 1A–H), as only 3.41 ± 1.12% of *DRD1* cells and 95.27 ± 0.93% of *DRD2* cells expressed *ENK. SubP* showed a preference for colocalization with *DRD1* (*z* = −5.424, *p* < 0.001), as 92.33 ± 1.49% of *DRD1* cells expressed *SubP*, while only 2.32 ± 0.51% of *DRD2* cells expressed *SubP* (Supplementary Figures 2A–H). *DRD1, DRD2*, and *A2aR* were expressed evenly in all striatal areas (Figures 1G,H). Finally, *DRD1* was expressed in 33.75 ± 1.02% of all striatal cells, while *DRD2* was expressed in 25.17 ± 0.82% of striatal cells, which was significantly less (*z* = −4.748, *p* < 0.001) (Figure 1H).

**Table 2:** **Percentage of coexpression of all investigated mRNAs with *Drd1* or *Drd2* transcripts**.

**Gene**	**Expression with *Drd1* (%)**	**Expression with *Drd2* (%)**
*A2aR*	0.39 ± 0.15	98.72 ± 0.33
*CB1R*	39.765 ± 7.34	36.66 ± 7.07
*DOR*	0.78 ± 0.31	70.27 ± 6.30
*DRD1*	100 ± 0.00	0.14 ± 0.01
*ENK*	3.41 ± 1.12	95.27 ± 0.93
*MC3R*	None observed	None observed
*MC4R*	12.93 ± 3.25	10.94 ± 2.45
*MOR*	8.30 ± 1.66	3.70 ± 1.01
*KOR*	25.34 ± 2.54	6.38 ± 1.19
*SubP*	92.33 ± 1.49	2.32 ± 0.51

### Coexpression of *DRD1*, *DRD2* and cannabinoid receptor 1

Striatal areas differentially expressed mRNA transcripts of the cannabinoid receptor 1 (*CB1R*) (χ^2^ = 11.984, *df* = 4, *p* = 0.017), as the highest percentage of *CB1R* positive cells was found in the lCPu (55.05 ± 2.61%), while lower amounts of *CB1R* positive cells were observed in the other parts of the striatum (mCPU: 20.65 ± 6.15%, AccC: 12.30 ± 1.91%, AccSh: 13.85 ± 3.17% and OT: 9.5 ± 1.40%), forming a decreasing dorsolateral to ventromedial gradient (Figure 2G). The *CB1R* showed no preference for coexpression with mRNAs of either of the two dopaminergic GPCRs, as 39.765 ± 7.34% of *DRD1* expressing cells and 36.66 ± 7.07% of *DRD2* cells expressed *CB1R* (Figures 2A–C,E,F,H). In addition, the amount of *CB1R* expression per cell was also dependent on striatal area, as high expressing cells were predominantly found in the lCPu and low expressing cells in other parts of the striatum (Figures 2I–M). Also here, the *DRD1* and *DRD2* populations were segregated, as we found that only 0.20 ± 0.07% of *DRD1* expressing cells also expressed *DRD2* (*z* = −5.929, *p* < 0.001) (Figure 2H). Moreover, significantly more striatal cells expressed *DRD1* (34.91 ± 1.24%) than *DRD2* (23.17 ± 0.84%) (*z* = −5.195, *p* < 0.001) (Figure 2G).

### Coexpression of *DRD1* and *DRD2* with melanocortin receptors

No mRNA expression of the melanocortin receptor 3 (*MC3R*) was found (Supplementary Figure 3), but a clear signal was present for mRNAs of the melanocortin receptor 4 (*MC4R*) (Figure 3C) and *MC4R* was differentially expressed in striatal areas (χ^2^ = 17.361, *df* = 4, *p* = 0.002). The highest expression was found in the OT (21.43 ± 3.84%), when compared to the AccC (8.30 ± 1.83%) and AccSh (5.68 ± 1.63%). No *MC4R* expression was observed in the mCPu and virtually no expression in the lCPu (0.43 ± 0.24%), so *MC4R* mRNA expression gradually decreases ventral to dorsal parts of the striatum (Figures 3G,I–M). The *MC4R* showed no apparent preference for either dopaminergic GPCR, as 12.93 ± 3.25% of *DRD1* expressing cells and 10.94 ± 2.45% of *DRD2* expressed *MC4R* (Figures 3A–C,E,F,H), although it was observed that cells expressing high levels of *MC4R* also expressed *DRD1*, but not *DRD2* (Figures 3N–R). Again, the *DRD1* and *DRD2* populations were segregated, as 0.14 ± 0.08% of *DRD1* expressing cells also expressed *DRD2* (*z* = −6.054, *p* < 0.001) (Figure 3H). In addition, striatal cells expressed significantly more *DRD1* (34.35 ± 1.39%) than *DRD2* (20.23 ± 1.03%) (*z* = −5.384, *p* < 0.001) (Figure 3G).

### Coexpression of *DRD1* and *DRD2* with opioid receptors

**Figure 4:**
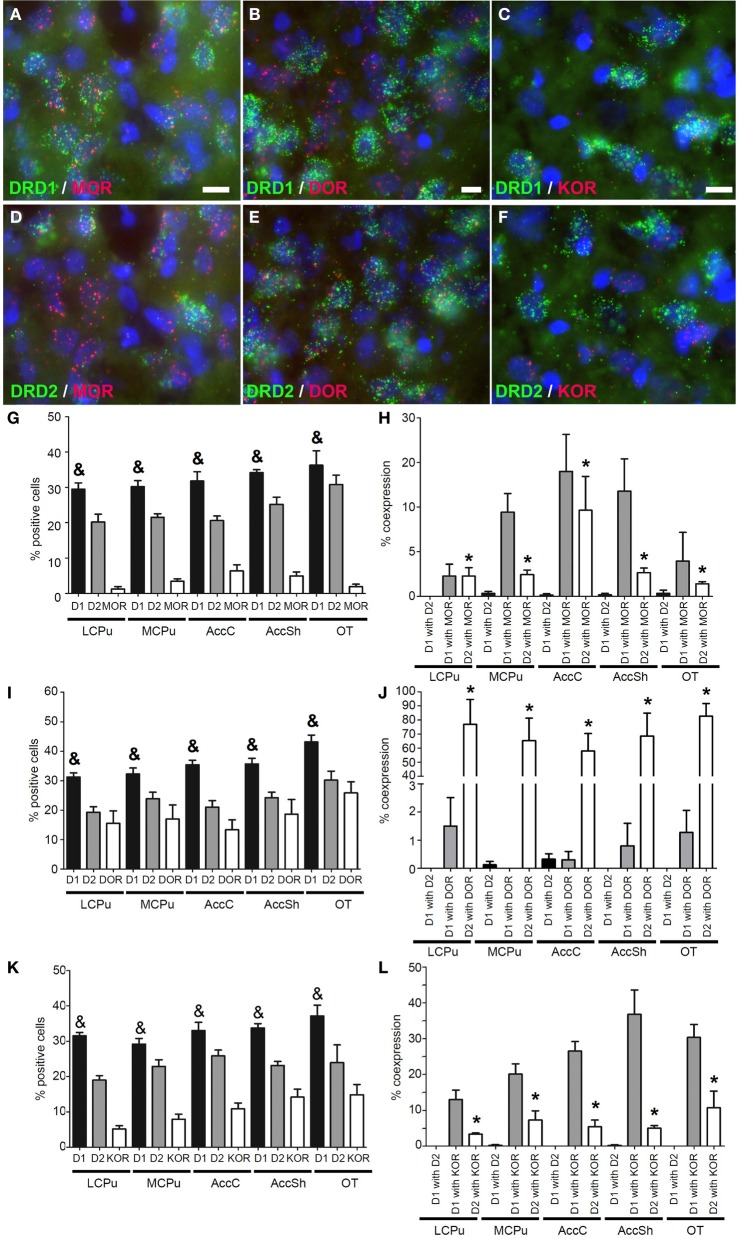
**Coexpression of the opiate receptors with both *DRD1* and *DRD2*. (A–F)** Example of staining in the striatum. Nuclei were stained using DAPI (blue). Coexpression depicted in red and green for the described receptors. White bar, 25 μm. **(A,D)** Coexpression for *DRD1* and mu-opioid receptor (*MOR*), also for *DRD2* and *MOR*. **(B,E)** Coexpression for *DRD2* and delta-opioid receptor (*DOR*), none found for *DRD1* and *DOR*. **(C,F)** Coexpression for *DRD1* and kappa-opioid receptor (*KOR*), and some coexpression for *DRD2* and *KOR*. **(G,I,K)** Quantification of coexpression of *DRD1*, *DRD2, MOR, DOR* and *KOR*. Bars represent the mean percentage (+s.e.m.) of DAPI stained nuclei that are positive for *DRD1, DRD2, MOR, DOR*, or *KOR*, where “&” indicates a significant difference of *p* < 0.05 in the mean percentage of *DRD1* positive cells compared to *DRD2* positive cells (as determined by the Mann–Whitney U test). **(H,J,L)** Quantification of coexpression of *DRD1* or *DRD2* and *MOR, DOR*, or *KOR*. Bars represent the mean percentage (+s.e.m.) of cells expressing *DRD1* also expressing *DRD2*, the percentage of *DRD1* expressing cells also expressing *MOR, DOR*, or *KOR* and the percentage of *DRD2* expressing cells also expressing *MOR, DOR*, or *KOR*, where “^*^” indicate significant differences of *p* ≤ 0.05 in the percentage of cells that coexpress *DRD1* and *MOR, DOR* or *KOR* compared to cells that coexpress *DRD2* and *MOR, DOR*, or *KOR* (as determined by the Mann–Whitney U test).

Also mRNA transcripts of the μ-opioid receptor (*MOR*) were differentially expressed within the striatum (lCPu: 1.225 ± 0.82%, mCPu: 3.45 ± 0.82%, AccC: 6.375 ± 1.99%, AccSh: 4.95 ± 1.28% and OT: 1.9 ± 0.85%) (χ^2^ = 10.928, *df* = 4, *p* = 0.027) (Figure 4G). *MOR* showed a preference for expression with the *DRD1*, as 8.30 ± 1.66% of *DRD1* expressing cells and 3.70 ± 1.01% of *DRD2* expressing cells also expressed *MOR* (*z* = −2.112, *p* = 0.035) (Figures 4A,D,H). Transcripts of the δ-opioid receptor (*DOR*) were dispersed evenly in striatal areas (lCPu: 15.55 ± 4.81%, mCPu: 17.03 ± 5.47%, AccC: 13.33 ± 3.91%, AccSh: 18.63 ± 5.78% and OT: 25.85 ± 4.37%) (Figure 4I). *DOR* showed a preference for expression with *DRD2*, as 0.78 ± 0.31% of *DRD1* expressing cells and 70.27 ± 6.30% of *DRD2* expressing cells also expressed *DOR* (*z* = −5.530, *p* < 0.001) (Figures 4B,E,J). Transcripts of the κ-opioid receptor (*KOR*) positive cells were differentially expressed in the striatum (lCPu: 5.175 ± 1.07%, mCPu: 7.93 ± 1.69%, AccC: 10.90 ± 1.85%, AccSh: 14.18 ± 2.60% and OT: 14.85 ± 3.36%) (χ^2^ = 10.928, *df* = 4, *p* = 0.027), showing a gradual increase in expression from dorsolateral to ventromedial parts of the striatum (Figure 4K). Moreover, *KOR* showed a preference for expression with the *DRD1*, as 25.34 ± 2.54% of *DRD1* expressing cells and 6.38 ± 1.19% of *DRD2* expressing cells expressed *KOR* as well (*z* = −4.951, *p* < 0.001) (Figures 4C,F,L). Also here, the *DRD1* and *DRD2* populations were segregated, as 0.13 ± 0.04% of *DRD1* expressing cells also expressed *DRD2* (*z* = −7.554, *p* < 0.001) (Figure 3H). In addition, striatal cells did express significantly more *DRD1* (34.35 ± 1.39%) than *DRD2* (20.23 ± 1.03%) (*z* = −5.384, *p* < 0.001) (Figure 3G).

## Discussion

To the best of our knowledge, we here show for the first time which percentage of striatal cells express *DRD1* and *DRD2* mRNAs and transcripts of the cannabinoid, melanocortin or opioid receptors. We first investigated if the expression of genes that are known to be expressed in only one of the two MSN populations indeed segregate along these lines. The D1R population is known to express SubP, while the D2R population can be identified by expression of ENK (Gerfen and Young, 1988). Moreover, A2aR is known to inhibit the function of the D2R by the formation of a functional heterodimer (Canals et al., 2003; Azdad et al., 2009), for which coexpression is a prerequisite (Schiffmann et al., 1991). Indeed, we observed near specific coexpression of the transcripts of *SubP* with *DRD1* mRNAs and of *ENK* and *A2aR* transcripts with *DRD2* mRNAs.and found very few cells (<1%) expressing both *DRD1* and *DRD2*. In addition, we found that striatal cells express *DRD1* in higher percentages than *DRD2*, which confirmed earlier findings (Bertran-Gonzalez et al., 2008; Matamales et al., 2009; Gangarossa et al., 2013b).

As stated earlier, a clear distinction in dopaminergic GPCR expressing populations can be made based on the expression patterns of either the D1R or the D2R (Gerfen et al., 1990; Le Moine and Bloch, 1995). However, this clear segregation of the D1R and D2R populations has been subject of much debate (Bertran-Gonzalez et al., 2010), as a number of groups have reported *DRD1* and *DRD2* coexpressing cells and the existence of functional D1R/D2R heterodimers (Larson and Ariano, 1994; Shetreat et al., 1996; Aizman et al., 2000; Hasbi et al., 2009; Perreault et al., 2012). These studies should be interpreted with care however, as the use of striatal cultures, in which neurons are functioning in an artificial environment, as well as cross-reactivity of the used antibodies may explain the reported D1R/D2R heterodimers, In any case, we also found instances of *DRD1* and *DRD2* coexpression, in percentages similar to studies using BAC transgenic mice (Gangarossa et al., 2013b). Moreover, a small overlap of the D1R and D2R populations is also evidenced by our finding that the neuropeptides *ENK* and *SubP* are expressed in both MSN populations. In addition, a recent study suggests that an area between the AccSh and AccC is an ideal anatomical substrate where D1R/D2R signaling could take place (Gangarossa et al., 2013a). So, MSNs might not be completely segregated into a D1R/SubP population and a D2R/ENK/A2aR population, depending on striatal area. Future research has to show if and where the two MSN populations overlap and establish the functional consequences of this presumed overlap.

Our next focus was on the expression of *CB1R* in the two MSN populations. *CB1R* mRNA was highly expressed in the dorsolateral part of the striatum and its expression decreased toward ventromedial parts of the striatum, in congruence with earlier reports (Hermann et al., 2002; Martin et al., 2008). Extending on these results, we found that the *CB1R* is promiscuous in its coexpression with the dopaminergic GPCRs and that nearly all *DRD1* and *DRD2* positive neurons expressed the *CB1R* in the lCPu, while this percentage was much lower in the ventral striatum. Despite the lower expression of *CB1R* in the ventral parts of the striatum, our findings stress the ability of endocannabinoids to influence both motor functions and reward processing (Monory et al., 2007; Ferre et al., 2010). Moreover, our data confirm results of earlier studies that showed that the cannabinoid system is able to give local feedback on the activity of both MSN populations (for extensive review see: Fitzgerald et al., 2012)

The melanocortinergic system is well known for its role in the regulation of energy balance, in particular through the action on its two centrally expressed GPCRs: the *MC3R* and the *MC4R* (Gantz and Fong, 2003). No indication of striatal expression of *MC3R* transcripts was found, readily excluding a prominent role for *MC3R* signaling in the striatum. Our observations suggest that the melanocortinergic system primarily interacts with ventral parts of the striatum. Looking at the percentage of coexpression of *MC4R* transcripts with either *DRD1* or *DRD2* transcripts, no significant differences were observed. However, the expression levels of *MC4R* transcripts correlate with the expression of either the *DRD1* or the *DRD2*, as high levels of *MC4R* transcripts coincide with coexpression of *DRD1*, but not *DRD2* mRNAs. This correlation is of particular interest in light of a number of studies that reported the involvement of MC4Rs expressed on D1R MSNs in a range of behavioral phenotypes. Lim et al. have found that the MC4R mediates stress-induced decreases in the strength of excitatory synapses onto D1R, but not D2R expressing MSNs, as the MC4R is necessary to internalize AMPA receptors on D1R MSNs after chronic stress (Lim et al., 2012). Moreover, MC4Rs expressed on D1R MSNs mediate parts of the phenotype observed in MC4R null mice, such as increased meal size, insensitivity to cocaine-induced anorexia and locomotor sensitization and the inability to be conditioned for high-fat food reinforcers (Cui et al., 2012; Cui and Lutter, 2013). In addition, the high expression of MC4R on D1R MSNs could explain why the infusion of MC4R antagonists blocks the behavioral effects of cocaine, which are thought to be mediated primarily by D1R signaling (Hsu et al., 2005). Taken together, these findings indicate that MC4R signaling strongly modulates D1R signaling, making it an interesting target for pharmacotherapies to combat psychopathologies such as addiction and obesity.

Opioids generally act upon three different GPCRs. These GPCRs differ in opioid affinity and are expressed throughout the entire striatum (Mansour et al., 1995). We and others observed high *MOR* mRNA expression in the AccC and AccSh, implying a stronger influence of *MOR* on the nucleus accumbens. Moreover, we confirmed a gradual increase in the expression of *KOR* transcripts from dorsolateral toward ventromedial parts of the striatum (Mansour et al., 1994) and showed that all investigated opioid receptors show a preference for expression with either of the to dopaminergic GPCRs: the *DOR* showed near exclusive coexpression with the *DRD2*, the *KOR* showed a preference for coexpression with the *DRD1*, and the *MOR* showed a preference for coexpression with the *DRD1*.

The main agonist for DOR is enkephalin, for KOR dynorphin and for MOR endorphin (Corbett et al., 2006). Dynorphins and enkephalins are synthesized in DRD1 and DRD2 expressing neurons, respectively, (Gerfen et al., 1990), while endorphins are synthesized in POMC expressing neurons (Corbett et al., 2006). These observations led us to postulate that dynorphins and enkephalins are able to give selective feedback on DRD1 and DRD2 expressing neurons, respectively. Since opioid receptors signal via Gi resulting in hyperpolarization via activation of GIRK channels (Torrecilla et al., 2002), release of opioids from striatal MSNs may provide an auto-inhibitory feedback loop. KOR activation has been associated with dysphoria and aversion (Pfeiffer et al., 1986; Shippenberg et al., 2001), while DOR activation has been suggested to improve negative emotional states (Filliol et al., 2000; Ragnauth et al., 2001; Roberts et al., 2001; Perrine et al., 2006). Also, activation of DOR increases the release of dopamine in the striatum (Di Chiara and Imperato, 1988), while KOR activation has the opposite effect (Mulder et al., 1984). These opposing roles are also reflected in the differential influence of these opioid receptors on dopamine-related behaviors (Shippenberg and Herz, 1987; Bals-Kubik et al., 1989). It is tempting to speculate that this is due to the preference of these receptors for one of the two MSN populations, but our results are not congruent with results of others, which found DRD1 to coexpress with DOR in much higher levels than we did (Ambrose et al., 2006). This might be due to differences in subcellular localization at the pre- or post-synapse, to internalization of the receptor, to the striatal region that was under investigation in the other study (a specific part of the dorsal striatum) or to cross-reactivity of the antibodies used and future studies will have to clarify this discrepancy. Taken together, our observations suggest that *MOR* is able to modulate the activity of both MSN populations, with a stronger influence on the *DRD1* population, as opposed to *DOR* and *KOR*, which seem to target specific MSN subpopulations.

Although through the quantification of the percentage of striatal cells that express *DRD1* and *DRD2* mRNAs with transcripts of cannabinoid, melanocortin or opioid GPCRs this study provides valuable insight in the cellular architecture of the striatum, it has two major limitations that should be taken into account. Firstly, the nature of our methods do not allow for any inference on the location of the proteins that are translated from the GPCR mRNAs. As such, we cannot distinguish between pre- and postsynaptically located GPCRs and thus do not provide the exact location where endocannabinoids, melanocortins, and opioids act to modulate MSN activity. Secondly, we only used hemispheres of two different animals in this study. As the majority of our results are so prominent (the GPCRs under investigation showed either a clear preference for colocalization with the *DRD1* or *DRD2* or not), we could not justify increasing the amount of animals because of ethical reasons.

In sum, the present data indicate that the dopaminergic, cannabinoid, and opioid systems exert control over the entire striatum, while the melanocortinergic system specifically targets the ventral parts. Moreover, we find that the opioid GPCRs have a preference for expression with either the *DRD1* or *DRD2* and that high and low levels of *MC4R* expression imply coexpression with *DRD1* and *DRD2*, respectively. Hereby, we expand the list of GPCRs that are preferentially expressed in one of the MSN populations and offer some understanding of the molecular mechanisms that enable the selection of adaptive behaviors.

### Conflict of interest statement

The authors declare that the research was conducted in the absence of any commercial or financial relationships that could be construed as a potential conflict of interest.
