# Supplementary figures and images for: Cannabinoid, melanocortin and opioid receptor expression on DRD1 and DRD2 subpopulations in rat striatum
Source: Front Neuroanat. 2014 Mar 26;8:14. doi: 10.3389/fnana.2014.00014 (PMC3972466; doi:10.3389/fnana.2014.00014)

Supplemental Figure 1

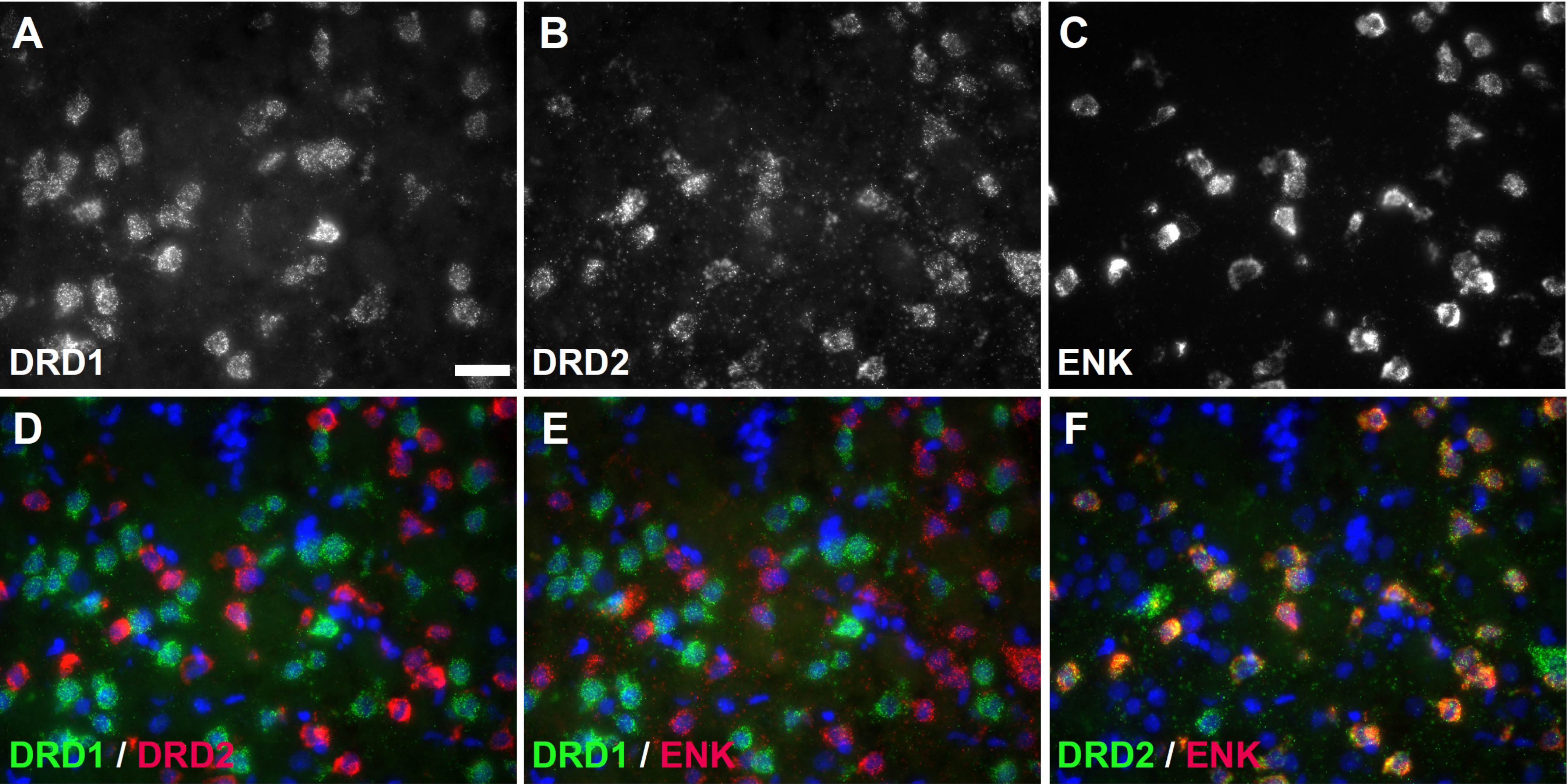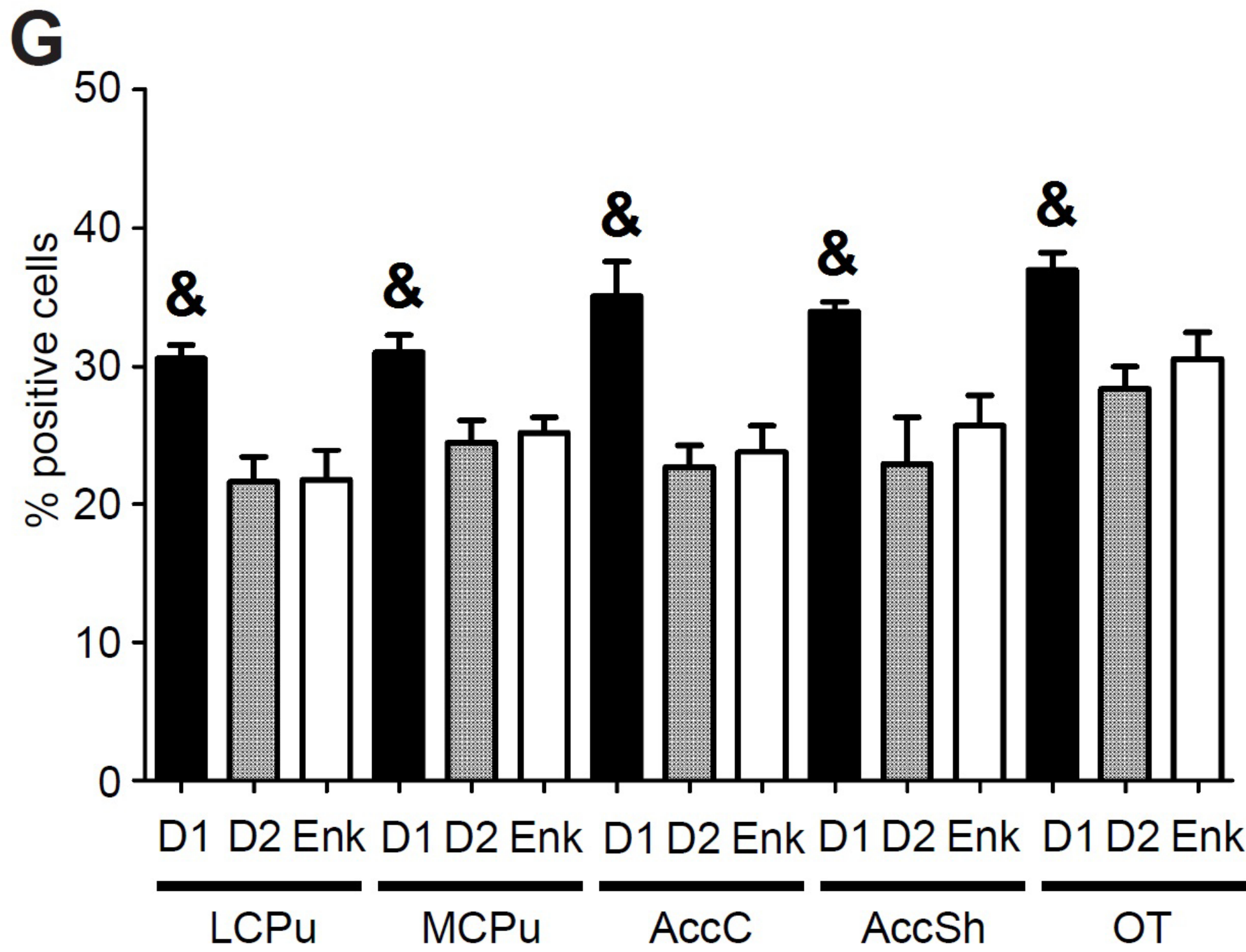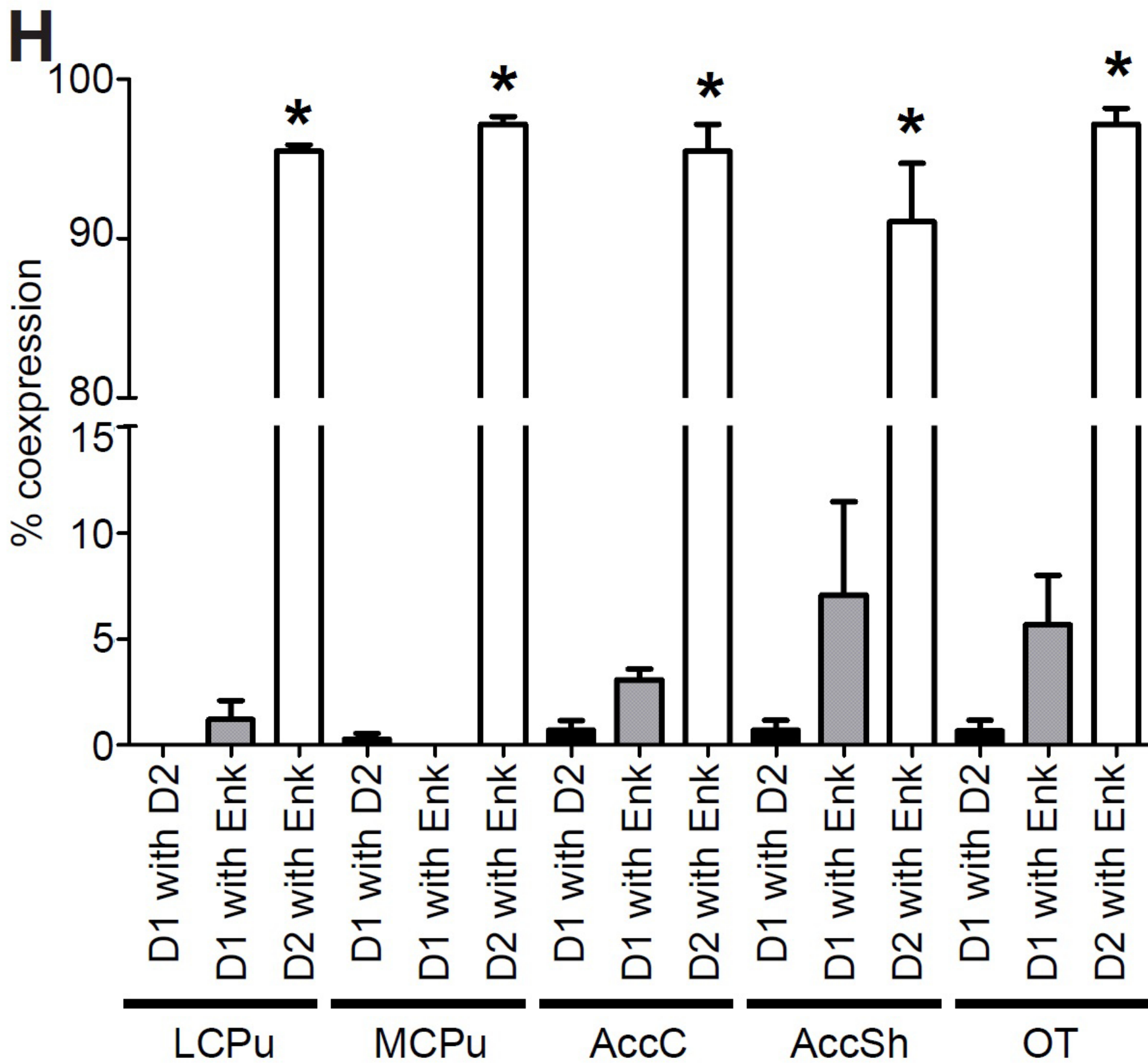

Supplement: Supplementary file 1 [file Presentation1.PDF]

Supplemental Figure 2

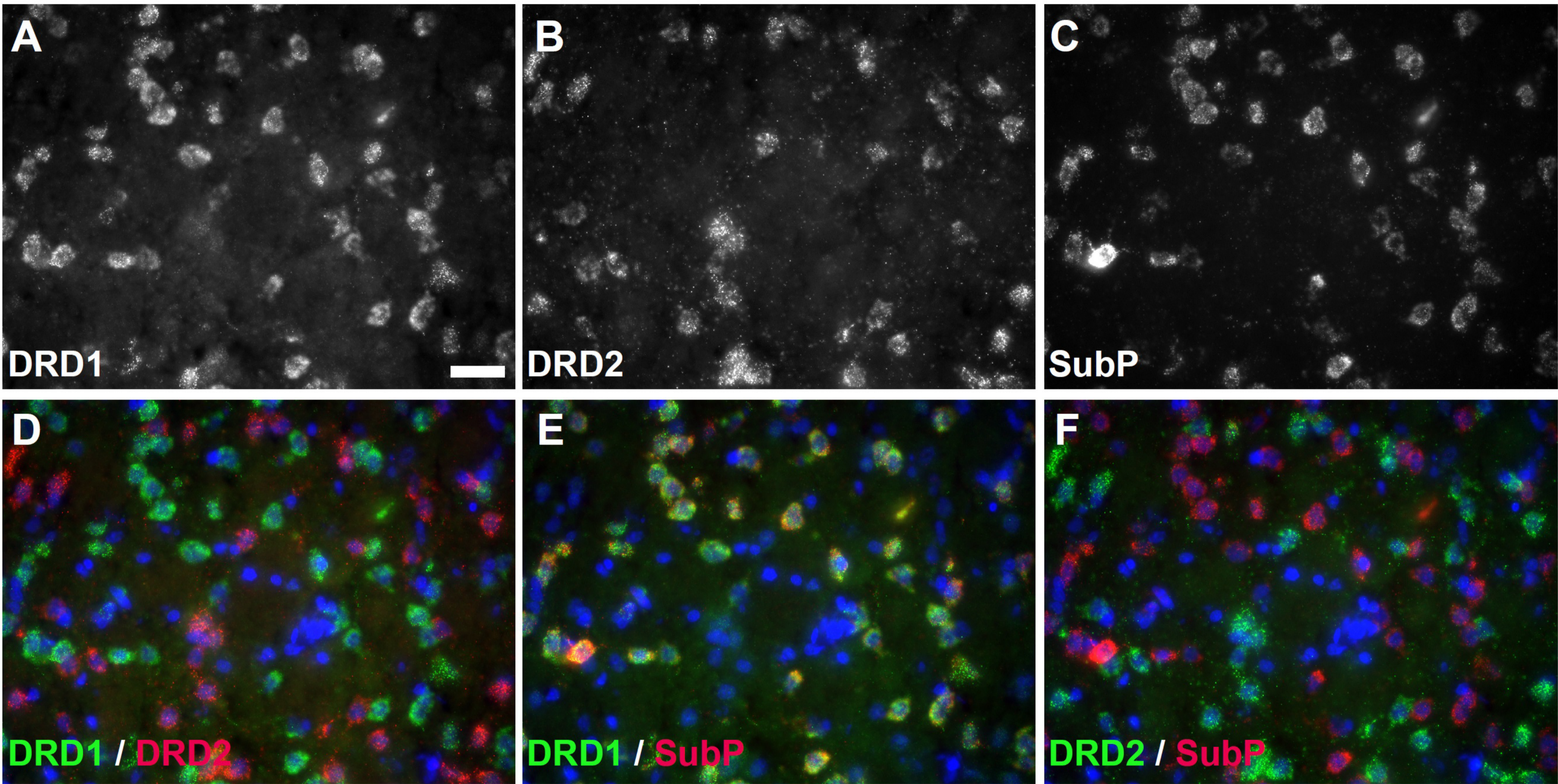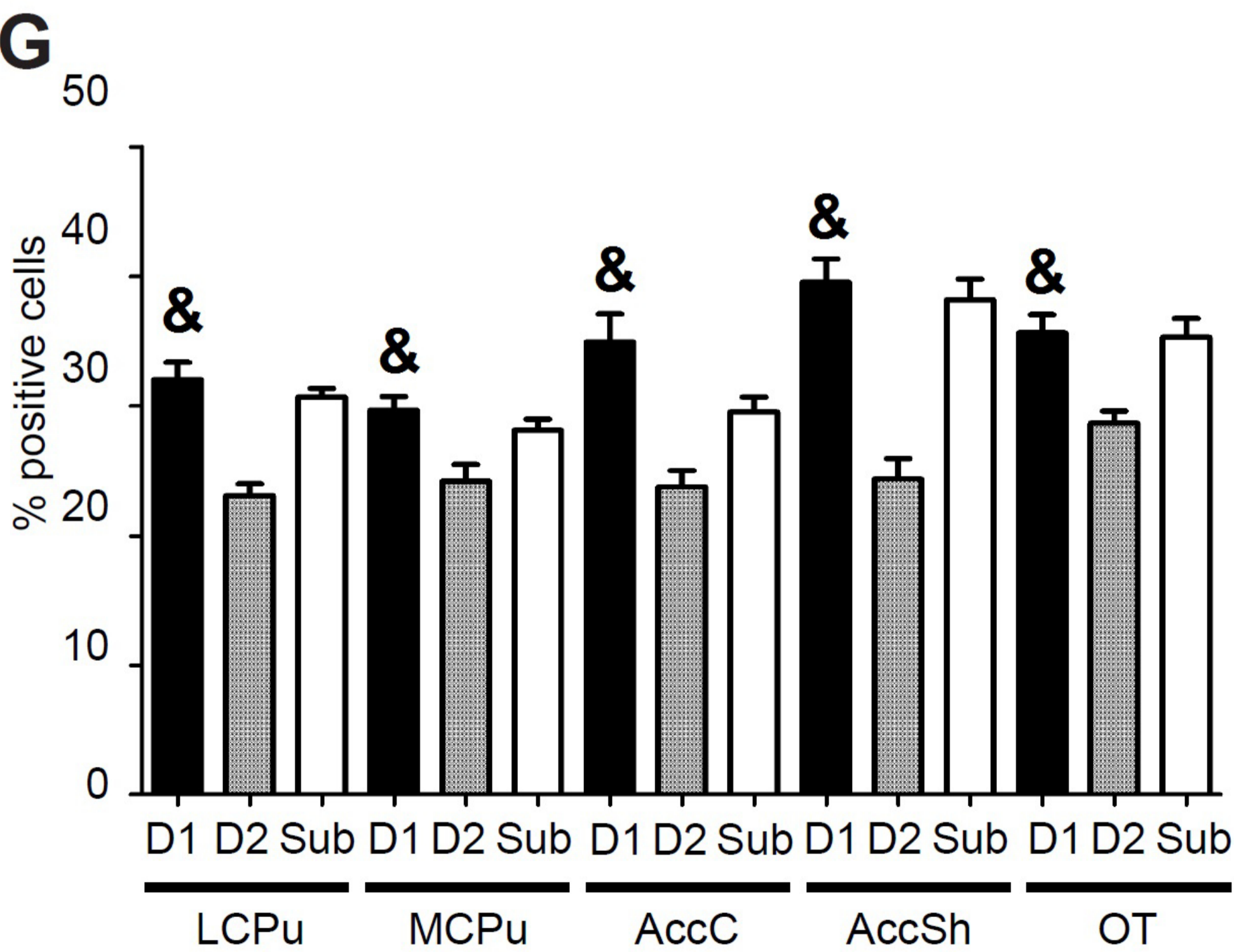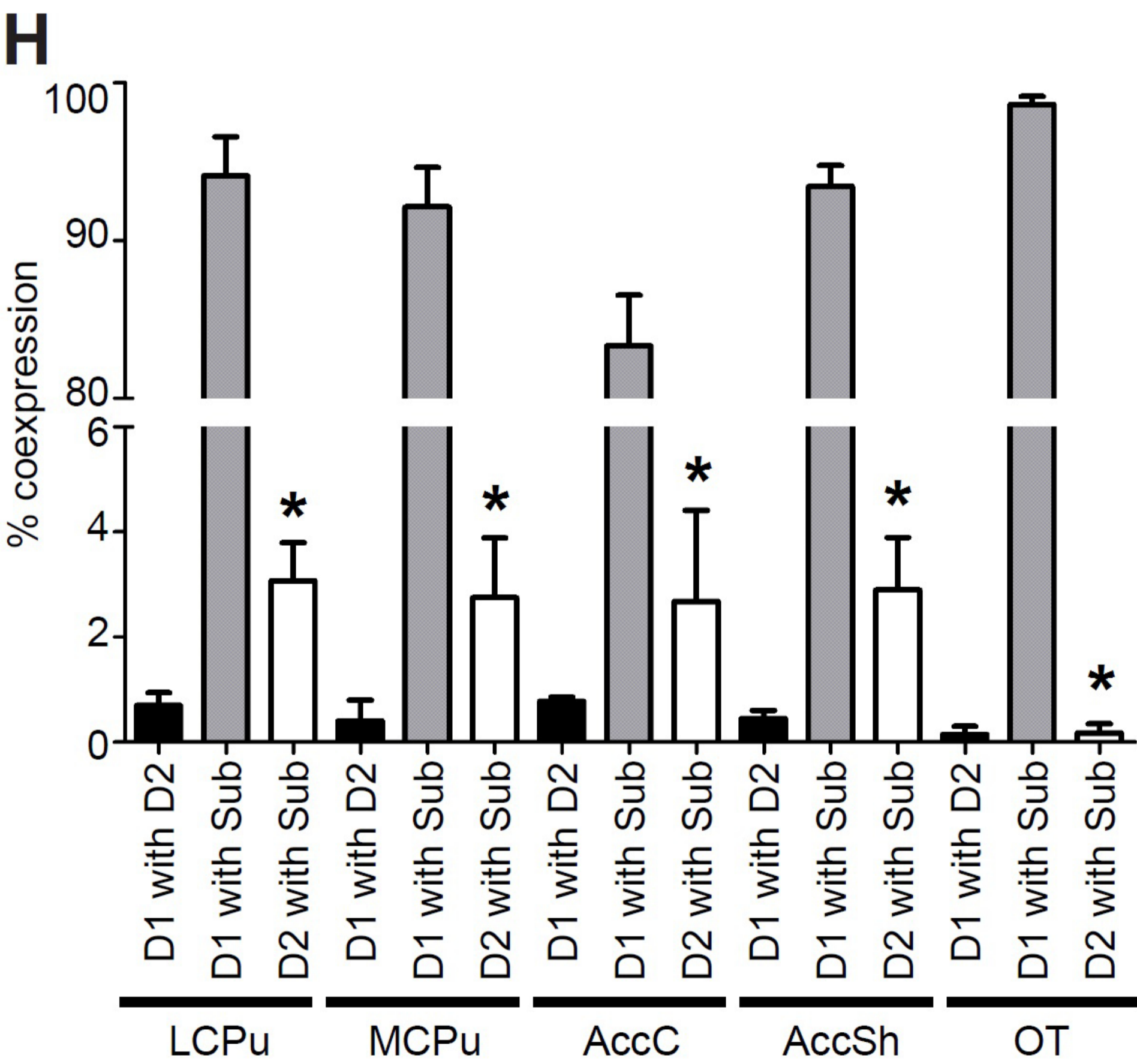

Supplement: Supplementary file 2 [file Presentation2.PDF]

# Supplemental Figure 3

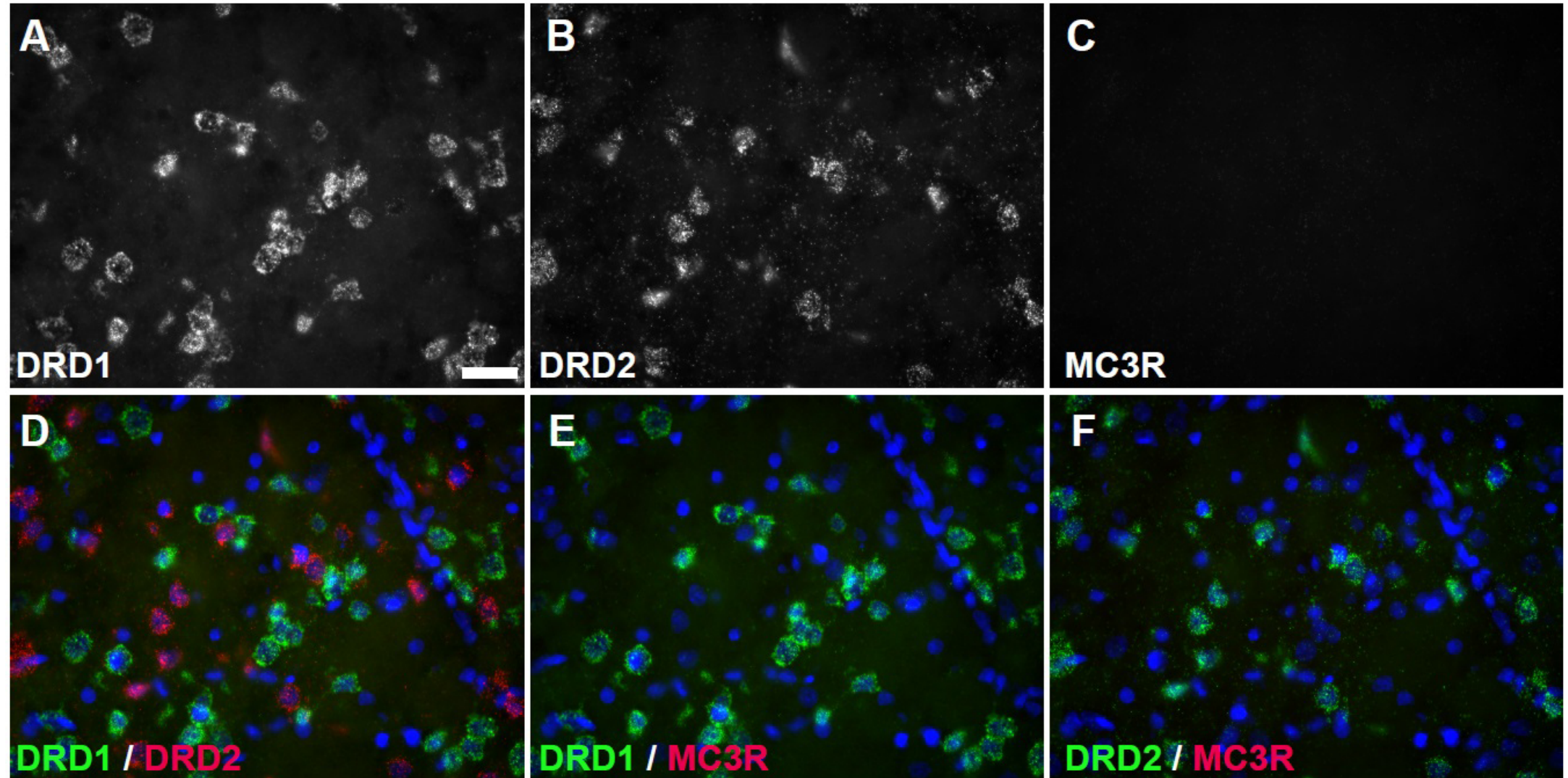

Supplement: Supplementary file 3 [file Presentation3.PDF]
